# Multidisciplinary Approach to Right Ventricular Myxoma

**DOI:** 10.21470/1678-9741-2020-0177

**Published:** 2021

**Authors:** Ali Karagöz, Berhan Keskin, Ahmet Karaduman, Seda Tanyeri, Taylan Adademir

**Affiliations:** 1 Department of Cardiology, Kartal Kosuyolu Research and Education Hospital, Kartal, İstanbul, Turkey.; 2 Department of Cardiovascular Surgery, Kartal Kosuyolu Research and Education Hospital, Kartal, İstanbul, Turkey.

**Keywords:** Cardiac Tumors (Incl Primary, Metastatic), Myxoma, Imaging (All Modalities; Specify Target)

## Abstract

Right ventricular (RV) myxoma that obstructs the RV outflow tract is rare. Multimodality imaging is crucial due to the curved and triangular shape of the RV anatomy. Incomplete resection by the right atrial approach in cardiac myxomas may be prevented by preoperative imaging with echocardiography, computed tomography and magnetic resonance imaging to provide detailed visualization. Right ventriculotomy may be an alternative approach to the isolated atrial approach to get complete resection of RV myxoma in suitable patients. The preferred surgical treatment is not well defined for ventricular myxomas and careful preoperative planning is essential. Surgical resection should be performed as soon as possible to avoid outflow tract obstruction, which might result in sudden death. The collaboration between cardiologist and heart surgeon and the effective use of imaging tools are essential for successful treatment. In this article, diagnosis and treatment and the heart team approach to RV myxoma are discussed with a demonstrative patient.

**Table:** 

Abbreviations, acronyms & symbols	
**CMR**	**= Cardiac magnetic resonance**
**CT**	**= Computed tomography**
**RV**	**= Right ventricle**
**2D-TTE**	**= Two-dimensional transthoracic echocardiography**

## INTRODUCTION

Cardiac myxomas are benign tumors of endocardial origin. Symptoms might mimic heart disease as well as infectious disease, immunodeficiency, and malignant processes. Myxomas arise from right ventricle (RV) in 3.7% of cases; however, myxomas originating in the RV free wall are rare and not a well-defined pathologic process in the literature^[1]^.

Due to the complex shape of the RV, is difficult to generate images of its components simultaneously by using two-dimensional transthoracic echocardiography (2D-TTE). Cardiac magnetic resonance (CMR) plays an important role in the evaluation of cardiac masses, especially when echocardiographic findings are suboptimal, or when the localization is atypical. The evaluation of regional wall motion abnormalities and tissue characterization are only possible with CMR. Multimodality imaging is an important concept for the diagnosis and treatment process. In this article, we present a patient with recurrent RV myxoma evaluated by multimodality imaging and successfully managed.

## TECHNIQUE

**Fig. 1:**
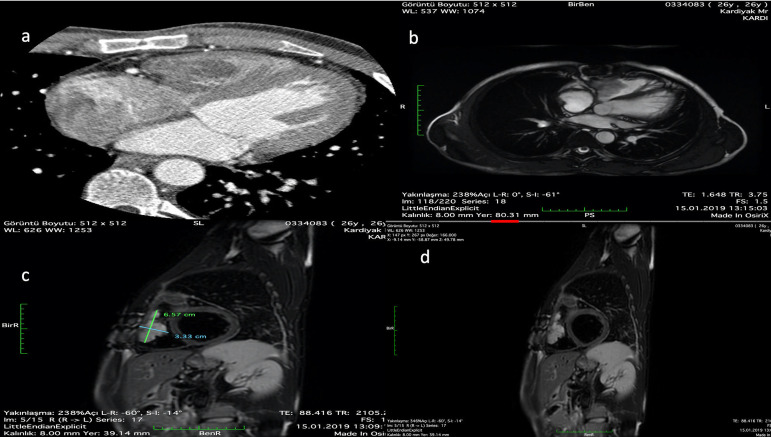
(A) Computed tomography shows right ventricular myxoma that is connected to right ventricular basal free wall. (B) Four-chamber MRI view shows right ventricular myxoma that is connected to the right ventricular basal free wall. (C) MRI short-axis view shows the measure of the myxoma in 6,57×3,33 centimeters. (D) Gadolinium-enhanced MRI imaging revealed heterogeneous delayed enhancement of the RV mass confirming the diagnosis of myxoma.

A 26-year-old-man was referred to our clinic with recurrent cardiac tumor, which was operated by atrial approach two months ago. We detected a large RV myxoma connected to the basal free wall and anterior leaflet of the tricuspid valve with 2-D TTE, CMR and computed tomography (CT) (Figure 1A and 1B, Video 1 to 3). CMR revealed a huge (6.57×3.33 centimeters) mobile heterogenous mass (Figure 1C). The 2-D TTE showed that a myxoma caused partial RV inflow obstruction (Video 4). Cardiac CT showed that the basal free wall of RV was invaded by the myxoma and the RV was mildly dilated (Figure 1B). Gadolinium-enhanced CMR imaging revealed heterogeneous delayed enhancement in RV mass, confirming the diagnosis of myxoma (Figure 1D).

**Video 1:**
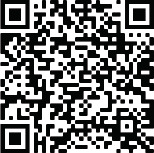
Two-dimensional transthoracic echocardiography, parasternal short axis view, shows right ventricular mass connected to the right ventricular basal free wall.

**Video 3:**
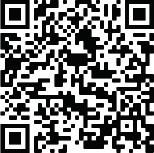
Two-dimensional transthoracic echocardiography shows right ventricular mass attached to the anterior leaflet of the tricuspid valve.

**Video 4:**
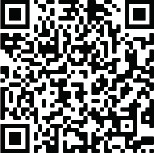
Two-dimensional transthoracic echocardiography color Doppler shows the systolic motion of the right ventricular mass and systolic turbulence, which means right ventricular outflow tract obstruction.

**Fig. 2:**
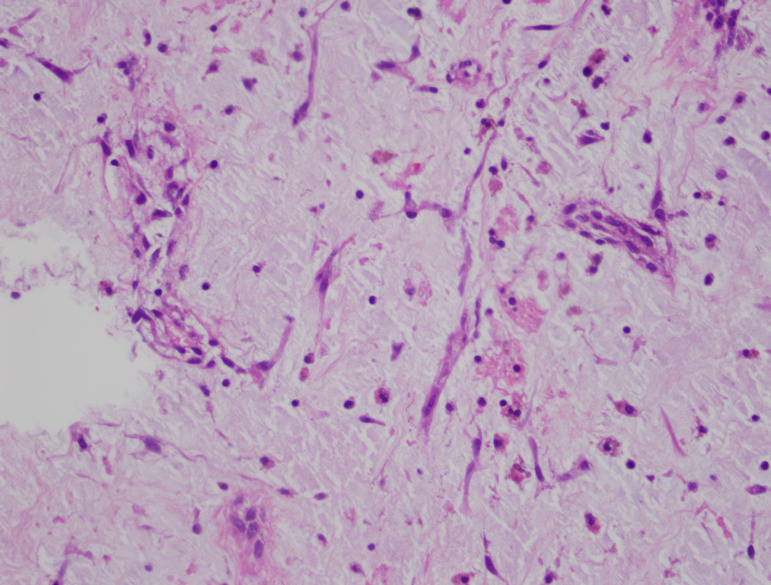
Postoperative histological specimen revealed myxoma cells, short strands embedded in the myxoid stroma.

The heart was exposed after re-sternotomy and release of adhesions. Aortic arterial and selective bicaval cannulation were done after systemic heparinization. Right atriotomy was performed under total cardiopulmonary bypass and exposed only part of the mass through the tricuspid valve. Thus, the tumor was exposed through a 4-cm long right ventriculotomy incision, perpendicular to the RV outflow tract, starting just below the pulmonary valve. The myxomatous mass that invaded the RV around the tricuspid valve, including the septal valve of the tricuspid valve and muscle of Lancisi, was exposed. The tumor in the RV muscle was excised with a 2-3 mm muscle tissue by using a scalpel. The tumor mass in the tricuspid valve and papillary muscle was scraped and cryoablation (AtriCure^TM^ cryoICE Cryoablation Probe at -60 ºC for 2 minutes) was used to naturalize the tumor cells in the valve and papillary muscles. In addition, cryoablation of the tumor base was performed, in our case, where the tumor was suspected, showing deep intramyocardial invasion. Right ventriculotomy was closed primarily with Teflon pledgets and Prolene sutures. Right atriotomy was closed primarily after stabilization of the tricuspid annulus with a De Vega annuloplasty suture. After surgery, imaging showed mild tricuspid regurgitation, with no residual tumor mass (Video 5 to 7). The histologic material obtained after operation was consistent with cardiac myxoma (Figure 2).

**Video 5:**
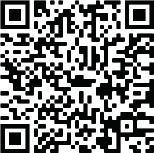
Postoperative two-dimensional transthoracic echocardiography short axis view shows the absence of a right ventricular mass.

**Video 6:**
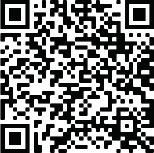
Postoperative two-dimensional transthoracic echocardiography four-chamber view shows the absence of a right ventricular mass

**Video 7:**
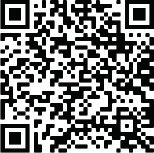
Postoperative color Doppler two-dimensional transthoracic echocardiography four-chamber view shows mild tricuspid regurgitation and absence of right ventricular outflow tract obstruction.

## DISCUSSION

Despite the low recurrence of myxoma with surgery, a large study showed that, in the univariate Cox regression model analysis, the right ventricular myxoma has a high recurrence with the only right atrial approach^[2]^. Although there is no clear explanation for a surgical approach in RV myxomas, right atrial surgical approach and the absence of detailed examination of the tumor's invasion site with multimodality imaging and the curved shape of the right ventricle may cause incomplete resection in our case^[3-5]^.

This case also highlights the need for noninvasive imaging modalities in the delineation of cardiac masses before contemplating surgical planning and the importance of heart team approach. Tumor seeding, incomplete removal, multiple tumor foci and malignancy can be causes of recurrence; however, insufficient preoperative imaging that causes inadequate surgery can be an important reason for treatment failure^[4,5]^.

## CONCLUSION

We used multimodality imaging and made preoperative planning with the heart team to provide surgical success. We decided on the right atrial and ventricular approach to provide complete resection. This experience underlines the importance of teamwork and detailed preoperative preparation. The use of both ventricular and atrial approach may be useful in the treatment of challenging RV myxomas.

**Table:** 

Authors' roles & responsibilities	
AK	Substantial contributions to the conception or design of the work; or the acquisition, analysis, or interpretation of data for the work; drafting the work or revising it critically for important intellectual content; final approval of the version to be published
BK	Substantial contributions to the conception or design of the work; or the acquisition, analysis, or interpretation of data for the work; drafting the work or revising it critically for important intellectual content; final approval of the version to be published
AK	Agreement to be accountable for all aspects of the work in ensuring that questions related to the accuracy or integrity of any part of the work are appropriately investigated and resolved; final approval of the versionto be published; final approval of the version to be published
ST	Agreement to be accountable for all aspects of the work in ensuring that questions related to the accuracy or integrity of any part of the work are appropriately investigated and resolved; final approval of the versionto be published; final approval of the version to be published
TA	Agreement to be accountable for all aspects of the work in ensuring that questions related to the accuracy or integrity of any part of the work are appropriately investigated and resolved; final approval of the versionto be published; final approval of the version to be published

## Figures and Tables

**Video 2:**
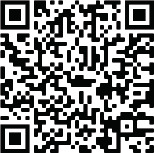
Four-chamber MRI view shows right ventricular mass connected to the right ventricular basal free wall.
